# Supplementary material for: Class 3 PI3K coactivates the circadian clock to promote rhythmic de novo purine synthesis
Source: Nat Cell Biol. 2023 Jul 6;25(7):975–88. doi: 10.1038/s41556-023-01171-3 (PMC10344785; doi:10.1038/s41556-023-01171-3)
Supplement: Supplementary file 1 — Reporting Summary [file 41556_2023_1171_MOESM1_ESM.pdf]

nature portfolio

Corresponding author(s): Dr. Ganna Panasyuk

Last updated by author(s): May 19, 2023

Reporting Summary

Nature Portfolio wishes to improve the reproducibility of the work that we publish. This form provides structure for consistency and transparency in reporting. For further information on Nature Portfolio policies, see our Editorial Policies and the Editorial Policy Checklist.

Statistics

For all statistical analyses, confirm that the following items are present in the figure legend, table legend, main text, or Methods section.

n/a	Confirmed	
☐	☒	The exact sample size (*n*) for each experimental group/condition, given as a discrete number and unit of measurement
☐	☒	A statement on whether measurements were taken from distinct samples or whether the same sample was measured repeatedly
☐	☒	The statistical test(s) used AND whether they are one- or two-sided<br>*Only common tests should be described solely by name; describe more complex techniques in the Methods section.*
☒	☐	A description of all covariates tested
☐	☒	A description of any assumptions or corrections, such as tests of normality and adjustment for multiple comparisons
☐	☒	A full description of the statistical parameters including central tendency (e.g. means) or other basic estimates (e.g. regression coefficient) AND variation (e.g. standard deviation) or associated estimates of uncertainty (e.g. confidence intervals)
☐	☒	For null hypothesis testing, the test statistic (e.g. *F*, *t*, *r*) with confidence intervals, effect sizes, degrees of freedom and *P* value noted<br>*Give P values as exact values whenever suitable.*
☒	☐	For Bayesian analysis, information on the choice of priors and Markov chain Monte Carlo settings
☒	☐	For hierarchical and complex designs, identification of the appropriate level for tests and full reporting of outcomes
☒	☐	Estimates of effect sizes (e.g. Cohen's *d*, Pearson's *r*), indicating how they were calculated

Our web collection on statistics for biologists contains articles on many of the points above.

Software and code

Policy information about availability of computer code

Data collection	Softwares for data collection are listed in Methods, including: ChemiDocTM Imager (BioRad) for Western Blots revelation; Zeiss Zen Blue 3.7 Microscopy Software was used for image processing following microscopy. For in vivo bioluminescence measurements, custom made lumicycler was used (Dr. Charna Dibner Laboratory as described in PMID: 33602874) and bioluminescence pattern was monitored by a LumiCycle 96 (Actimetrics).
Data analysis	The software packages used in this study are listed in the Methods, including: Western blot quantifications were done using ImageJ software (v. 2.1.0) and calculations in Microsoft Excel 365 (v. 2016) and GraphPad Prizm9. q-PCR data analyses were done using QuantStudio 1 (ThermoFisher Scientific) and calculations in Microsoft Excel 365 (v. 2016) and GraphPad Prizm9. Chip-seq and RNA-seq data were analyzed with SOAPnuke, Bowtie2 v2.4.4, SAMtools v 1.13, deepTools2, MACS2 v2.2.7.1, and R v4.1.2 (http://www.r-project.org/foundation/). Metabolite classes and pathways were identified using R package "MetaboAnalystR3.0". Rhythmicity was assessed with non-parametric "MetaCycle" (JTK_CYCLE) algorithm implemented in R. For data presentation, all data plots made with ggplot2 using R-studio. IGV_2.8.13 used for bigwig/bam file ChIP-seq visualization.

For manuscripts utilizing custom algorithms or software that are central to the research but not yet described in published literature, software must be made available to editors and reviewers. We strongly encourage code deposition in a community repository (e.g. GitHub). See the Nature Portfolio guidelines for submitting code & software for further information.

Data

Policy information about availability of data

All manuscripts must include a data availability statement. This statement should provide the following information, where applicable:

- Accession codes, unique identifiers, or web links for publicly available datasets
- A description of any restrictions on data availability
- For clinical datasets or third party data, please ensure that the statement adheres to our policy

All data are available in the main text or the supplementary materials. Deep-sequencing (ChIP-seq and RNAseq) data that support the findings of this study have been deposited in the Gene Expression Omnibus (GEO) under accession code GSE229551. Mass spectrometry metabolomics data are available as Supplementary data 6. Source data have been provided in Source Data file. All other data supporting the findings of this study are available from the corresponding author on reasonable request.

Human research participants

Policy information about studies involving human research participants and Sex and Gender in Research.

Reporting on sex and gender	NA
Population characteristics	NA
Recruitment	NA
Ethics oversight	NA

Note that full information on the approval of the study protocol must also be provided in the manuscript.

Field-specific reporting

Please select the one below that is the best fit for your research. If you are not sure, read the appropriate sections before making your selection.

☒ Life sciences ☐ Behavioural & social sciences ☐ Ecological, evolutionary & environmental sciences

For a reference copy of the document with all sections, see nature.com/documents/nr-reporting-summary-flat.pdf

Life sciences study design

All studies must disclose on these points even when the disclosure is negative.

Sample size	For the studies in cell lines we determined the number of the experimental repetitions based on our previous studies using these lines (pubmid: 26387534; 23630012). All experiments were independently three times unless specified in the legends, and mean and the standard error from the mean were calculated.<br>In studies with animals, the "n" number corresponds to an individual mouse. Power analysis was used to determine the animal numbers to take into account previously observed magnitude of response to deletion of gene of interest in vivo (e.g. metabolic parameters, autophagy block for Vps15 deletion) and observations of circadian behavior for C57/B6 strain of mice previously reported (expression and metabolomics analyses). We applied >80% power and error rate 5% (two sided type 1) to detect >1.5 effect.
Data exclusions	No data or animals were excluded from the analyses.
Replication	All in vitro and in vivo assays have been performed 3 times unless specified in the legends. The accompanying quantification and statistics were derived from n=3 independent replicates unless specified in the legends. For biological replicates, independent preparation of depleted cells (adenoviral infection) were used to ensure reproducibility. For in vivo experiments, 3 mice per condition were analyzed. The numbers of replicates and mice used for each analysis is indicated in figure legends.
Randomization	For studies in cells, the treatment groups were attributed randomly between plates and wells. For studies in vivo, animals, including those treated with tamoxifen for gene deletion, were randomly allocated into experimental groups based on genotype. The breeding was set up in order of obtain both genotypes in the same litter.
Blinding	The blinding was applied for quantifications of all image-based analyses. In all treatments in vitro and in vivo, the samples were number-coded and the investigators were blinded during extract preparations for molecular analyses (e.g. transcript and protein expression, luciferase assays). Depending on the analysis, the groups were revealed either at the stage of sample quantifications and statistical analyses (e.g. qPCR, luciferase assay) or when it was essential for analysis (e.g. immunoblot of time series to include all time points/genotype/treatments in the same gel panel, immunoprecipitation or ChIP to set-up control and experimental groups with antibodies).

Reporting for specific materials, systems and methods

We require information from authors about some types of materials, experimental systems and methods used in many studies. Here, indicate whether each material, system or method listed is relevant to your study. If you are not sure if a list item applies to your research, read the appropriate section before selecting a response.

Materials & experimental systems

n/a	Involved in the study
☐	☒ Antibodies
☐	☒ Eukaryotic cell lines
☒	☐ Palaeontology and archaeology
☐	☒ Animals and other organisms
☒	☐ Clinical data
☒	☐ Dual use research of concern

Methods

n/a	Involved in the study
☐	☒ ChIP-seq
☒	☐ Flow cytometry
☒	☐ MRI-based neuroimaging

Antibodies

Antibodies used

All antibodies are listed in Methods section: Vps15 (1:1000, Abnova, H00030849-M02; For Proximity ligation assay 1:200, Genetex, GTX108953), p62 (1:1000, Abnova, H00008878-MO1), β-actin (1:5000, Sigma, A5316), Tubulin (1:1000, Sigma, T9026), β-catenin (1:500, BD Biosciences, 610153), Lamin A/C (1:1000, Cell Signaling, 2032), LC3 (1:1000, NanoTools, 0231-100/LC3-3-5-5F10), GAPDH (1:1000, Santa Cruz, SC-25778), REV-ERBα (1:1000, Cell Signaling, 13418S), BMAL1 (for IHC 1:250 and for Chip 4µg/IP, Abcam, ab3350; for WB 1:1000 and IF 1:200, Cell Signaling, 14020S), CLOCK (1:1000, Cell Signaling, 5157), HIS-Tag (1:1000, Proteintech, 66005-12-Ig), IPOA5 (1:1000, Proteintech, 18137-1-AP), Cry1 (1:500, Origene, TA342728), RNA Pol II total (1:1000 Active Motif, 39097), RNA Pol II phospho-Serine5 (1:1000, Chromotek, 3E8-1), RNA Pol II CTD phospho-Serine5 (1:1000, Abcam, ab5408), Vps34 (1:1000, Cell Signaling, 4263), Flag (1:1000, Sigma, F1804), Hsp90 (1:1000, Proteintech, 13171-1-AP), Histone H3 (1:3000, Cell Signaling 4499), normal-IgG Rabbit (Cell Signaling, 3900), normal-IgG Mouse (Santa Cruz, sc-2025), anti-Rabbit IgG HRP-linked Antibody (1:5000, Cell Signaling 7074), Anti-Mouse IgG HRP-linked Antibody (1:5000, Cell Signaling 7076), Donkey anti-Rabbit IgG (H+L) Highly Cross-Absorbed Secondary Antibody, Alexa Fluor Plus 488 (1:200, ThermoFisher, A32790), Donkey anti-Mouse IgG (H+L) Highly Cross-Absorbed Secondary Antibody, Alexa Fluor Plus 488 (1:200, ThermoFisher, A32766), Donkey anti-Rabbit IgG (H+L) Highly Cross-Absorbed Secondary Antibody, Alexa Fluor Plus 568 (1:200, ThermoFisher, A10042), Donkey anti-Mouse IgG (H+L) Highly Cross-Absorbed Secondary Antibody, Alexa Fluor Plus 568 (1:200, ThermoFisher, A10037), Goat anti-Rat IgG (H+L) Highly Cross-Absorbed Secondary Antibody, Alexa Fluor Plus 647 (1:200, ThermoFisher, A-21247).

Validation

The validation information for the antibodies used in this study are available on the websites of the respective commercial providers as well as in published studies. No custom-made antibodies were used in this study. In addition, for anti-Vps15, anti-Vps34 and anti-Bmal1 antibodies, the specificity in WB, IP and IF assays was validated in our laboratory in human and mouse cell models upon knock-down (siRNA or shRNA)/KO and overexpression of respective cDNA constructs.
The molecular weight markers were used to identify proteins of the expected molecular weight for the target proteins.

The links to the manufacturer's website for used antibodies could be found below:
Vps15 Abnova - Validated in WB, IF, IHC and ELISA: https://www.abnova.com/products/products_detail.asp?catalog_id=H00030849-M02

Vps15 Genetex - Validated in WB, KD/KO, Protein overexpression: https://www.genetex.com/Product/Detail/PI3-kinase-p150-antibody-C1C3/GTX108953

p62 Abnova - Validated in WB, ELISA, IF, IHC, IP: https://www.novusbio.com/products/p62-sqstm1-antibody-2c11_h00008878-m01

B-Actin Sigma - Validated in IHC, ELISA, IF, WB: https://www.sigmaaldrich.com/FR/en/product/sigma/a5441

Tubulin Sigma - Validated in IF and WB: https://www.sigmaaldrich.com/FR/en/product/sigma/t9026

B-Catenin BD Biosciences - Validated in WB, IF, IHC, IP: https://www.bdbiosciences.com/en-fr/products/reagents/microscopy-imaging-reagents/immunofluorescence-reagents/purified-mouse-anti-catenin.610153

Lamin A/C Cell Signaling - Validated for WB and IHC: https://www.cellsignal.com/products/primary-antibodies/lamin-a-c-antibody/2032

Histone H3 Cell Signaling - Validated for WB, IHC, IF, Flow Cyt: https://www.cellsignal.com/products/primary-antibodies/histone-h3-d1h2-xp-rabbit-mab/4499

LC3 Nanotools - Validated for WB, ICC, IHC: https://www.labome.com/product/Nanotools/0231-100-LC3-5F10.html

GAPDH Santa Cruz- Validated for WB, IHC, IF: https://www.scbt.com/p/gapdh-antibody-fl-335

Rev-Erba Cell Signaling - Validated for WB, IP, ChIP: https://www.cellsignal.com/products/primary-antibodies/rev-erba-e1y6d-rabbit-mab/13418

Bmal1 Abcam - Validated for WB, ICC: https://www.abcam.com/products/primary-antibodies/bmal1-antibody-ab3350.html

Bmal1 Cell Signaling - Validated for WB, IP, ChIP: https://www.cellsignal.com/products/primary-antibodies/bmal1-d2l7g-rabbit-mab/14020

Clock Cell Signaling - Validated for WB and IP: https://www.cellsignal.com/products/primary-antibodies/clock-d45b10-rabbit-mab/5157

His-Tag Proteintech - Validated for WB, IP, IF: https://www.ptglab.com/products/His-Tag-Antibody-66005-1-Ig.htm

IPOA5 Proteintech - Validated for WB, ELISA, IP, IHC, IF: https://www.ptglab.com/products/KPNA1-Antibody-18137-1-AP.htm

Cry1 Origene - Validated for WB and IHC: https://www.origene.com/catalog/antibodies/primary-antibodies/ta342728/cryptochrome-i-cry1-rabbit-polyclonal-antibody

RNA Pol II Active Motif - Validated for ChIP, WB, IF, ICC: https://www.activemotif.com/catalog/details/39097/rna-pol-ii-antibody-mab

RNA Pol II pSer5 Chromotek - Validated for WB and ChIP: https://www.citeab.com/antibodies/2452247-3e8-rna-pol-ii-ser5-p-antibody-3e8

RNA Pol II pSer5 Abcam - Validated for WB, ChIP, ELISA, ICC, Dot Blot, Flow Cyt. IF: https://www.abcam.com/products/primary-antibodies/rna-polymerase-ii-ctd-repeat-ysptsps-phospho-s5-antibody-4h8-chip-grade-ab5408.html

Vps34 Cell Signaling - Validated for WB and IP: https://www.cellsignal.com/products/primary-antibodies/pi3-kinase-class-iii-d9a5-rabbit-mab/4263

Flag Sigma - Validated for WB, IP, IF, ICC, IHC: https://www.sigmaaldrich.com/FR/en/product/sigma/f1804?gclid=Cj0KCQjw9deiBhC1ARIsAHLjR2AI28S1O1XODfRmBg7nS1dpYv_1hvWPg695SGJdQtx_7Kbk3iCCnkMaAmzhEALw_wcB&gclsrc=aw.ds

HSP90 Proteintech - Validated for WB, IP, IHC, IF, Flow Cyt, ELISA: https://www.ptglab.com/products/HSP90-Antibody-13171-1-AP.htm

IgG Rabbit Cell Signaling - Validated for WB, IF, IHC: https://www.cellsignal.com/products/primary-antibodies/rabbit-da1e-mab-igg-xp-isotype-control/3900

IgG Mouse Santa Cruz - Validated for WB, IHC, IF: https://www.scbt.com/p/normal-mouse-igg

Eukaryotic cell lines

Policy information about cell lines and Sex and Gender in Research

Cell line source(s)	HEK293T (CRL-3216) and AML12 (CRL-2254) cells were acquired from ATCC. Mouse embyonic fibroblasts (MEFs) were generated by our laboratory from Vps15f/f and Vps34 f/f mice. MEFs were obtained from a pool of embryos from two different females and were passaged every three days before spontaneous transformation. The MEF line proliferating after p25 was considered spontaneously immortalized.
Authentication	All members of our laboratory are trained on the Best Laboratory Practices and safe experimenting including cell line culture in order to prevent contamination with different cell types (ICLAC guidelines). HEK293T and AML12 cell lines were obtained from ATCC and were not further authenticated. For MEFs Vps15f/f or Vps34f/f knock-out was verified by western blots following Cre infections in every experiment.
Mycoplasma contamination	All cell lines used were bi-weekly tested for contaminations with mycoplasma using commercial PCR Mycoplasma Detection Kit (ABM, #G-238). All tests were negative.
Commonly misidentified lines (See ICLAC register)	No misidentified lines were used in this study

Animals and other research organisms

Policy information about studies involving animals; ARRIVE guidelines recommended for reporting animal research, and Sex and Gender in Research

Laboratory animals	Liver specific Vps15 knockout mouse line AlbCre+;Vps15f/f was derived from the strain #022624 (Jackson Laboratory, USA) crossed with AlbCre+ mice as reported (Nemazanyy et al., Nature Comm, 2015). Inducible hepatocyte-specific Vps15 knockout TtrCre+;Vps15 f/f line was generated by our laboratory as described in the methods sections.<br>Animals of 5 week old for AlbCre+;Vps15f/f and 12-16 week old for TtrCre+;Vps15f/f were used for the experimentation. All animals used in the study were fed ad libitum standard chow diet (Teklad Global 2918, 18% protein, irradiated) and kept under 12h/12h (8am/8pm) light on/off cycle under an ambient temperature (+21-22C) and humidity ranging between 50-60%. Animals were sacrificed at indicated time points in text, figures and figure legends. All animal studies were performed by authorized users in compliance with ethical regulations for animal testing and research.

Wild animals	No wild animals were used.
Reporting on sex	Male and female mice were used in this study as it is specified in figure legends.
Field-collected samples	The study did not involve samples collected in the field.
Ethics oversight	The study was approved by the ethical committee of University Paris Cité (authorization number APAFIS#32312 and APAFIS#14968).

Note that full information on the approval of the study protocol must also be provided in the manuscript.

ChIP-seq

Data deposition

☒ Confirm that both raw and final processed data have been deposited in a public database such as GEO.

☒ Confirm that you have deposited or provided access to graph files (e.g. BED files) for the called peaks.

Data access links<br>*May remain private before publication.*	The data are deposited in the Gene Expression Omnibus (GEO) under accession code GSE229551
Files in database submission	ChIP-Seq files:<br><br>ChIP_Bmal1_Vps15LKO_rep1.bed<br>ChIP_Bmal1_Vps15LKO_rep2.bed<br>ChIP_Bmal1_WT_rep1.bed<br>ChIP_Bmal1_WT_rep2.bed<br>ChIP_H3K27ac_Vps15LKO_rep1.bed<br>ChIP_H3K27ac_Vps15LKO_rep2.bed<br>ChIP_H3K27ac_WT_rep1.bed<br>ChIP_H3K27ac_WT_rep2.bed<br>ChIP_Vps15_rep1.bed<br>ChIP_Vps15_rep2.bed<br>Input_WT.bed<br>Input_Vps15LKO.bed<br><br>ChIP_Bmal1_Vps15LKO_rep1.fq.gz<br>ChIP_Bmal1_Vps15LKO_rep2.fq.gz<br>ChIP_Bmal1_WT_rep1.fq.gz<br>ChIP_Bmal1_WT_rep2.fq.gz<br>ChIP_H3K27ac_Vps15LKO_rep1.fq.gz<br>ChIP_H3K27ac_Vps15LKO_rep2.fq.gz<br>ChIP_H3K27ac_WT_rep1.fq.gz<br>ChIP_H3K27ac_WT_rep2.fq.gz<br>ChIP_Vps15_rep1.fq.gz<br>ChIP_Vps15_rep2.fq.gz<br>Input_WT.fq.gz<br>Input_Vps15LKO.fq.gz
Genome browser session<br>(e.g. UCSC)	Not applicable.

Methodology

Replicates	n=2 mice per condition were sequenced and analyzed.
Sequencing depth	Average of 30million (100bp-Paired End) clean reads following filtering low quality reads, N reads, and adapter sequences.
Antibodies	Vps15 Abnova, H00030849-M02. Bmal1 Abcam, ab3350. H3K27ac Cell Signaling, #8173.
Peak calling parameters	ChIP-Bmal1 with the following parameter: callpeak -t -c -f BAM -g mm -p 0.01<br>H3K27ac peak calling was performed using: callpeak -t -c -f BAM -g mm –broad<br>Vps15 was performed using: callpeak -t -c -f BAM -g mm -p 0.001 --broad –nomodel
Data quality	Data filtering was performed with SOAPnuke to remove adaptor sequences and low-quality reads. Data filtering parameter was: SOAPnuke filter -l 5 -q 0.5 -n 0.1 -Q 2 -c 40.
Software	Sequencing was performed with the DNBSEQ-400 sequencer (Beijing Genomics Institute, China). All data were analyzed with the pipeline: Bowtie2 for alignment to mm10, SAMtools for indexing and sorting SAM files, MACS2 for peak calling, R package Diffbind (V3.10.0) for differential peak calling, R package ChIPseeker and clusterProfiler for peak annotation and GO/KEGG pathway enrichment. Motif analysis was performed using HOMER, (findMotifsGenome.pl -mask -size 200). IGV_2.8.13 used for bigwig/bam file visualization.
