# Supplementary material for: Class 3 PI3K coactivates the circadian clock to promote rhythmic de novo purine synthesis
Source: Nat Cell Biol. 2023 Jul 6;25(7):975–88. doi: 10.1038/s41556-023-01171-3 (PMC10344785; doi:10.1038/s41556-023-01171-3)

**Uncropped blots Fig. 1.** Red square indicates the section shown in the main figures. Molecular weight is shown on the left.

b.

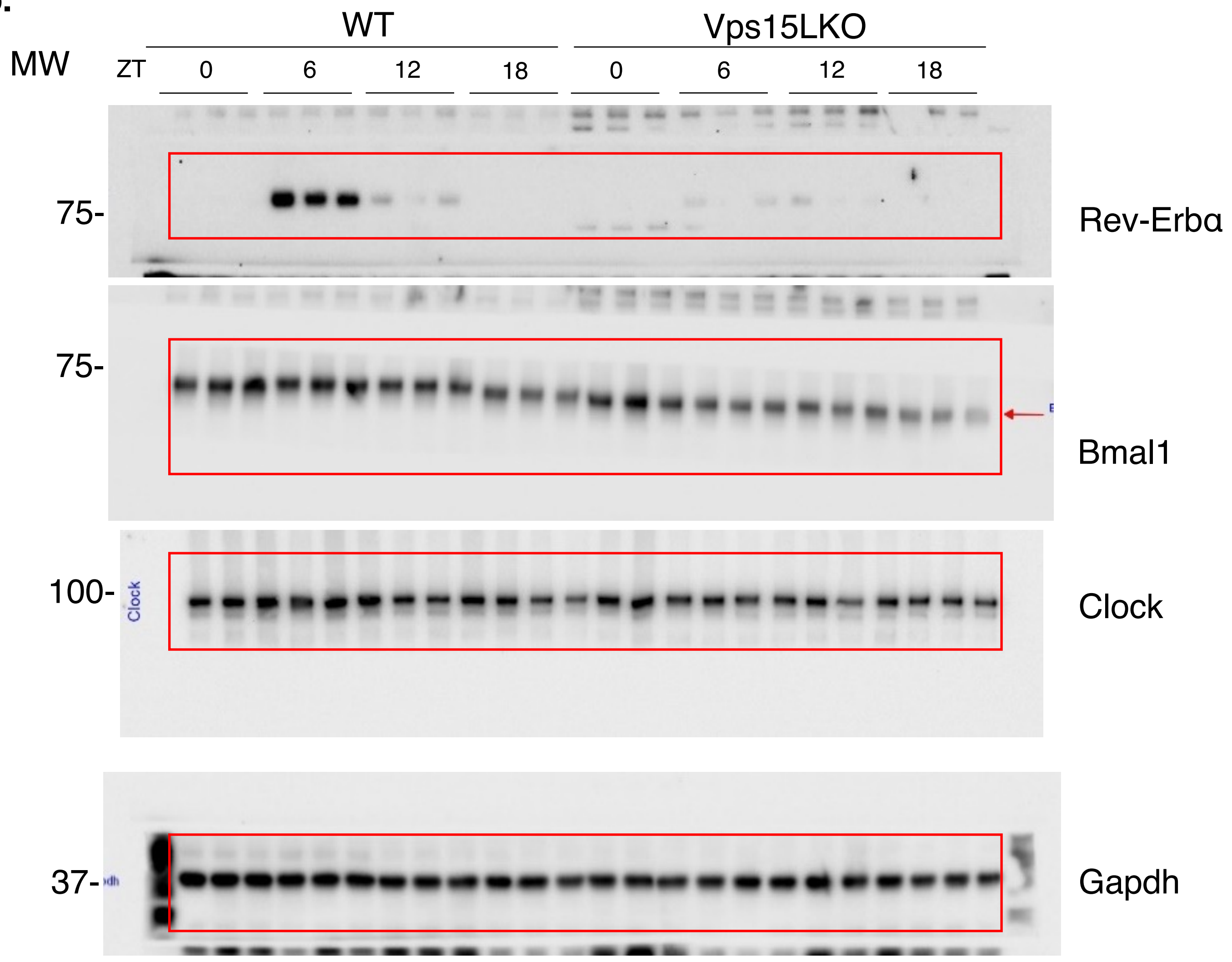


g.

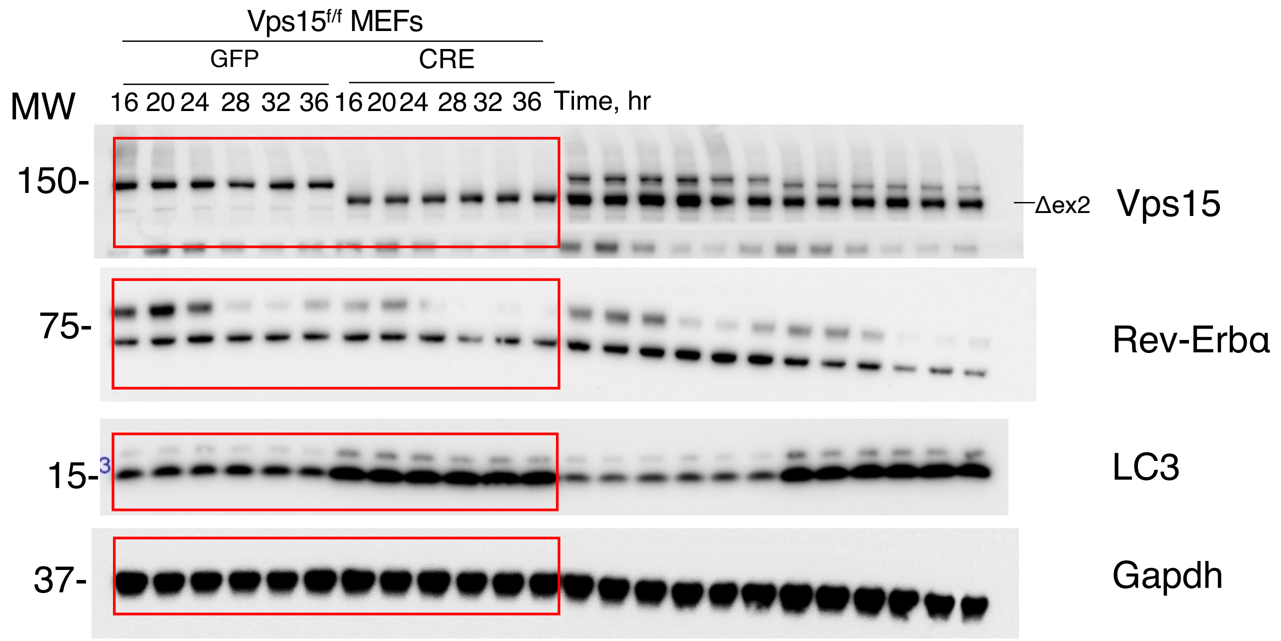

**Uncropped blots Fig. 2.** Red square indicates the section shown in the main figures. Molecular weight is shown on the left.

a.

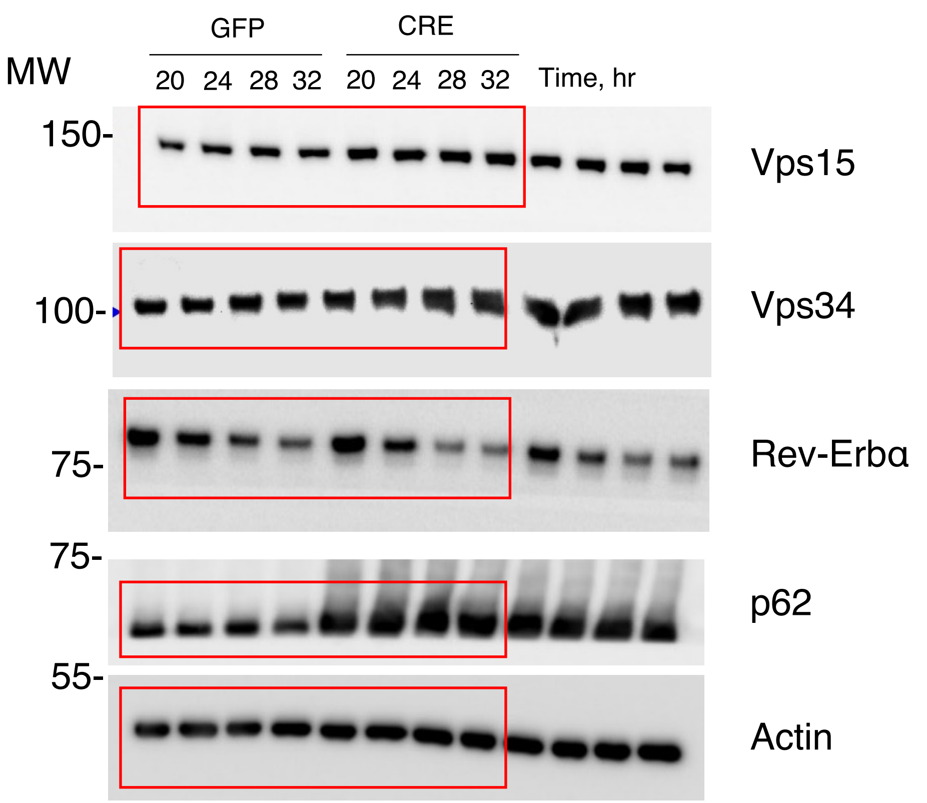


d.

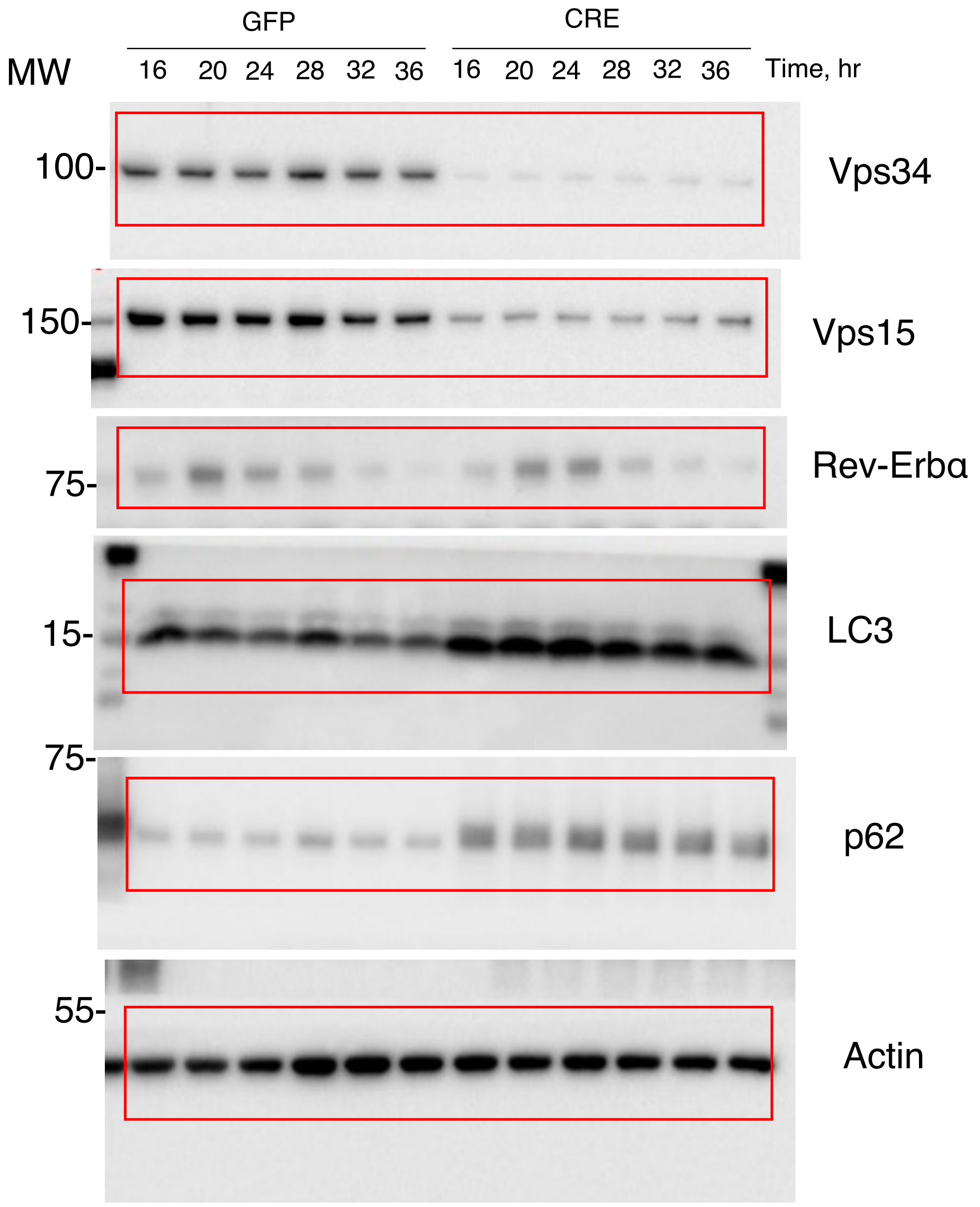

**Uncropped blots Fig. 3.** Red square indicates the section shown in the main figures. Molecular weight is shown on the left.

a.

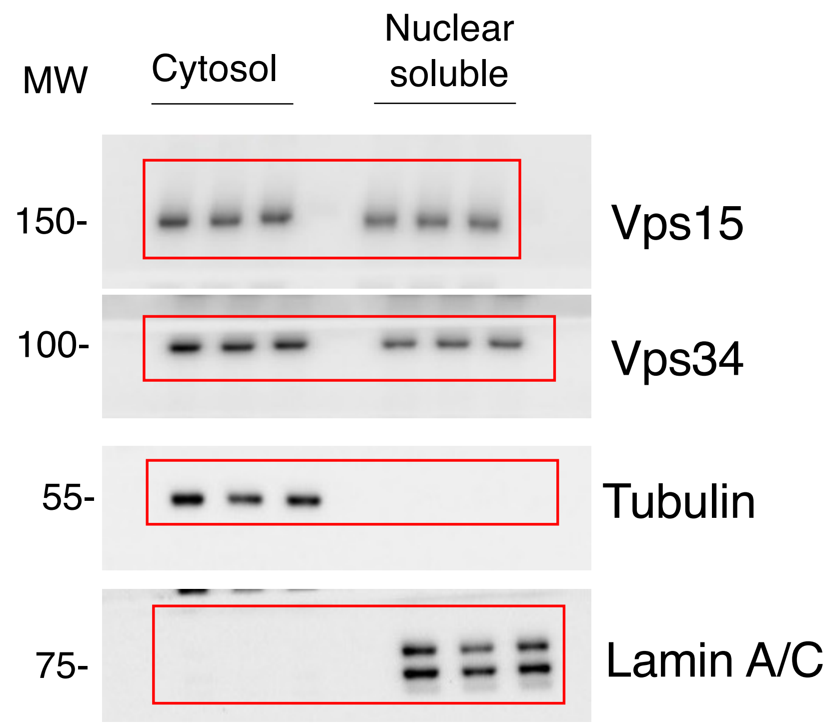


d.

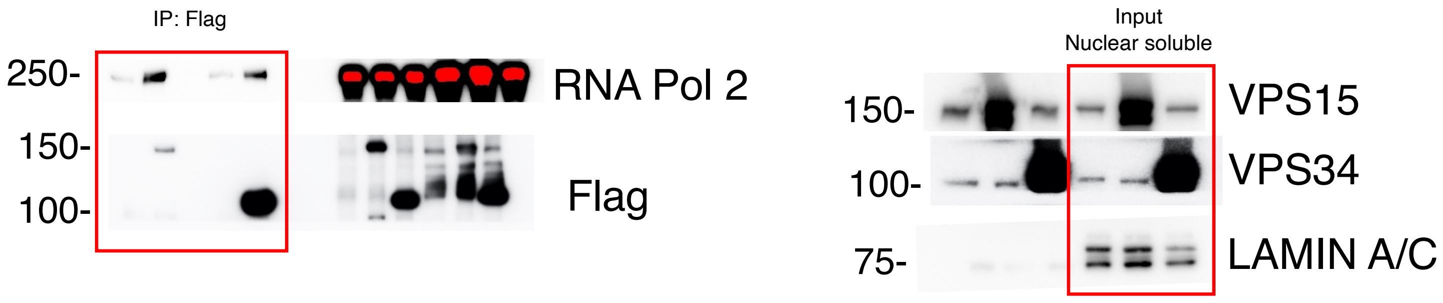


f.

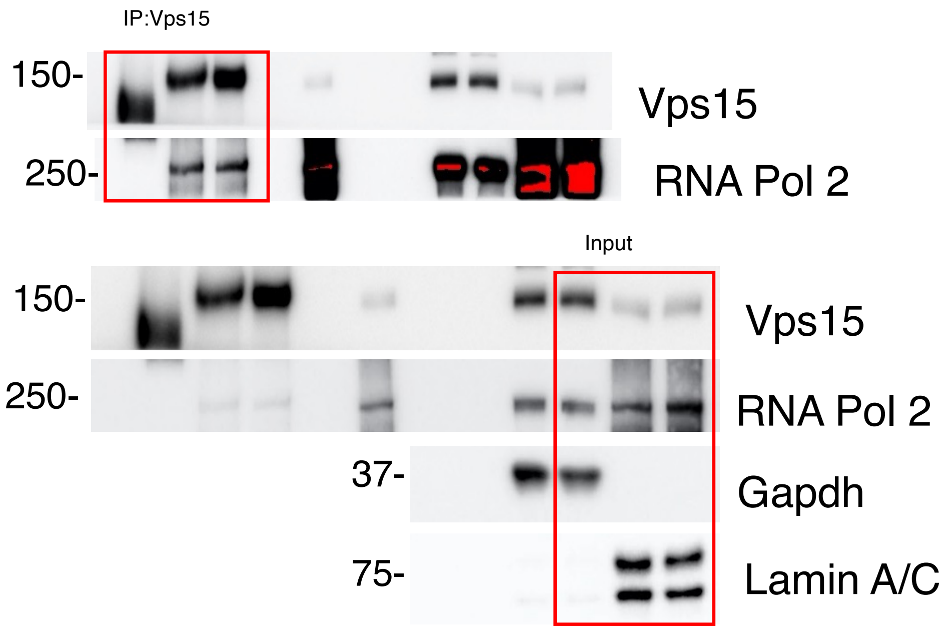


g.

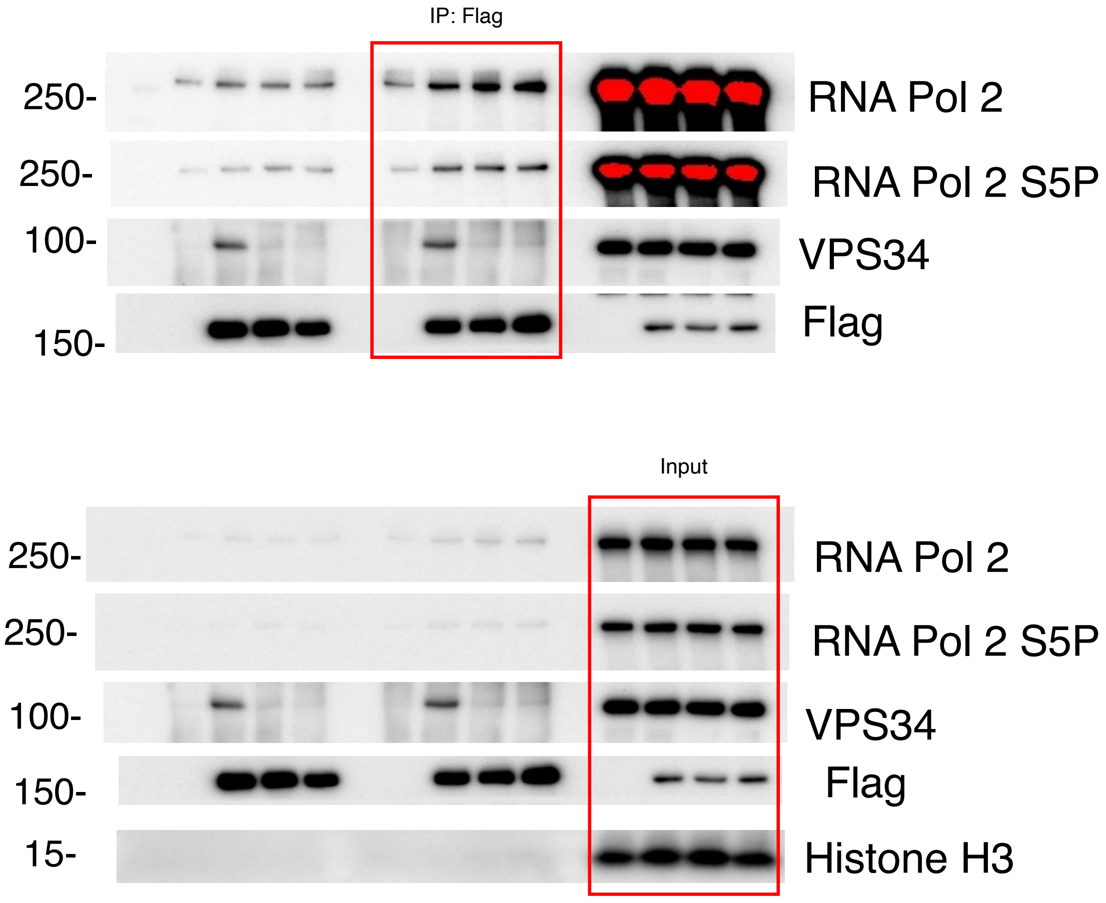

**Uncropped blots Fig. 4.** Red square indicates the section shown in the main figures. Molecular weight is shown on the left.

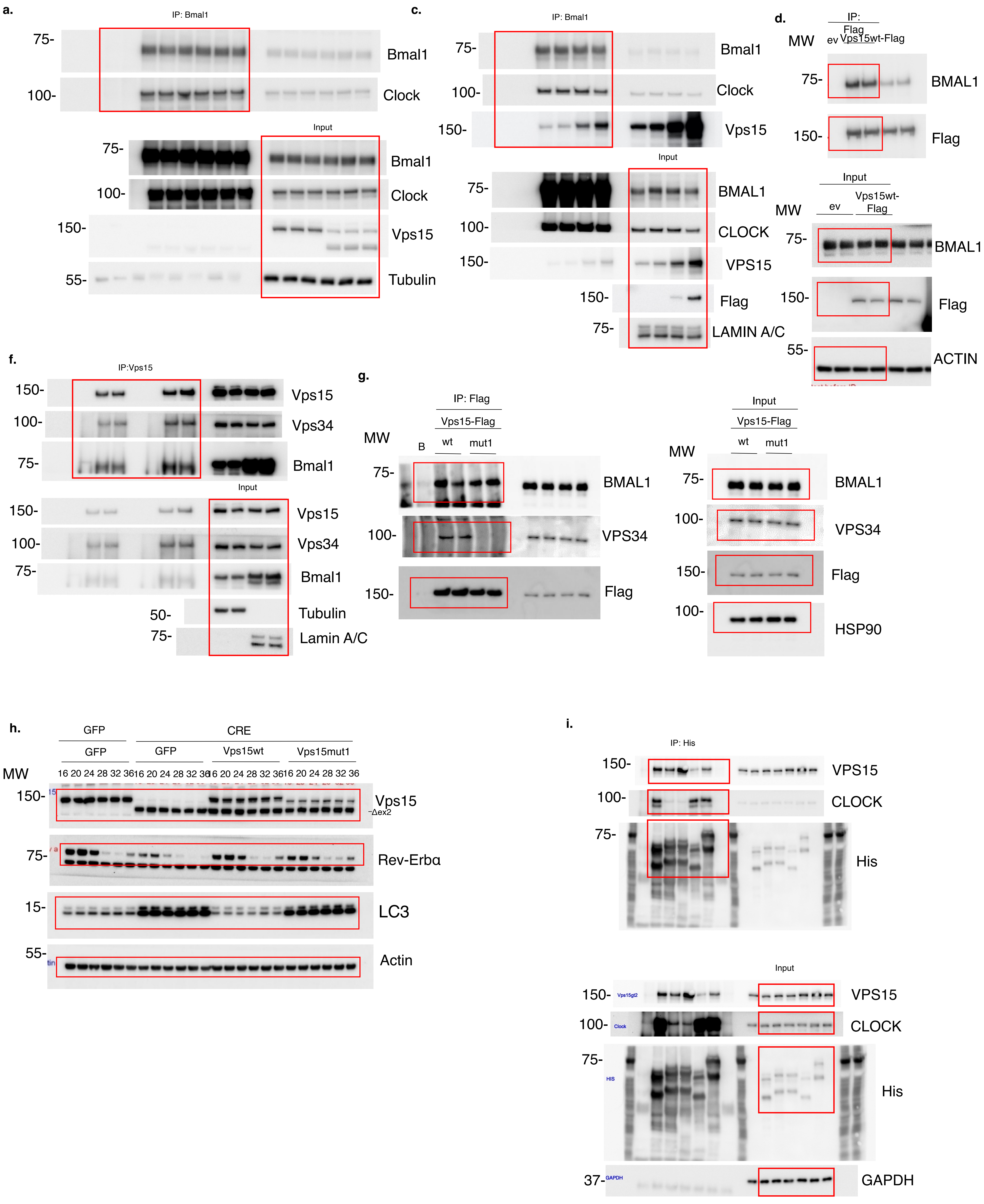

**Uncropped blots Fig. 5.** Red square indicates the section shown in the main figures. Molecular weight is shown on the left.

a.

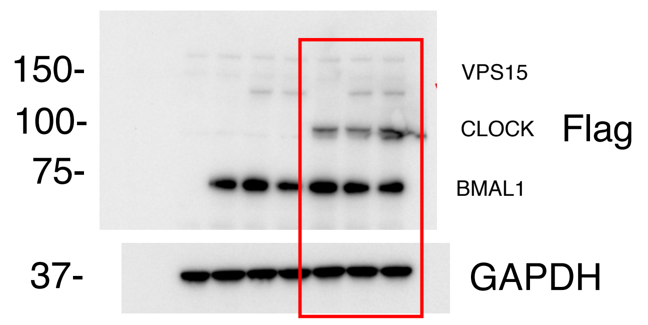


b.

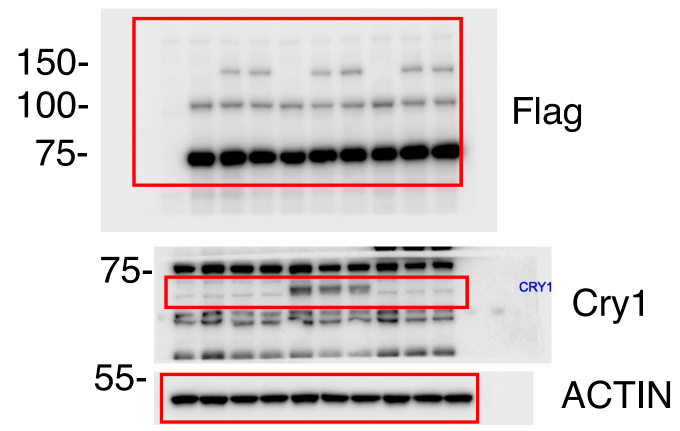

**Uncropped blots Fig. 6.** Red square indicates the section shown in the main figures. Molecular weight is shown on the left.

**f.**

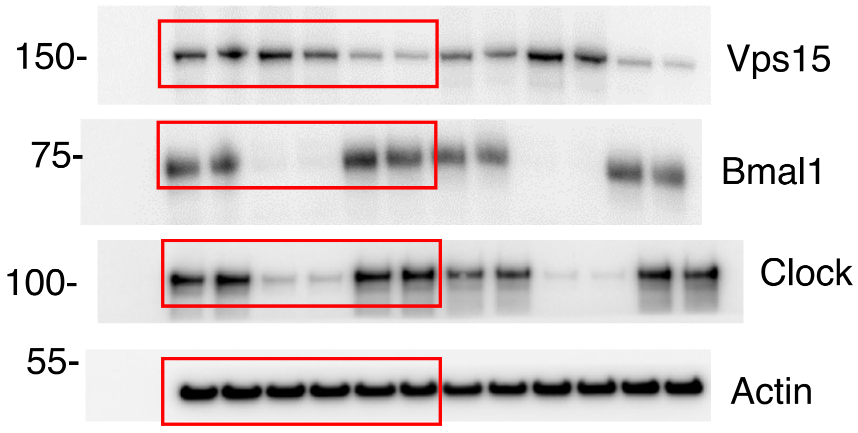

**Uncropped blots Extended Data Fig. 1.** Red square indicates the section shown in the main figures. Molecular weight is shown on the left.

a.

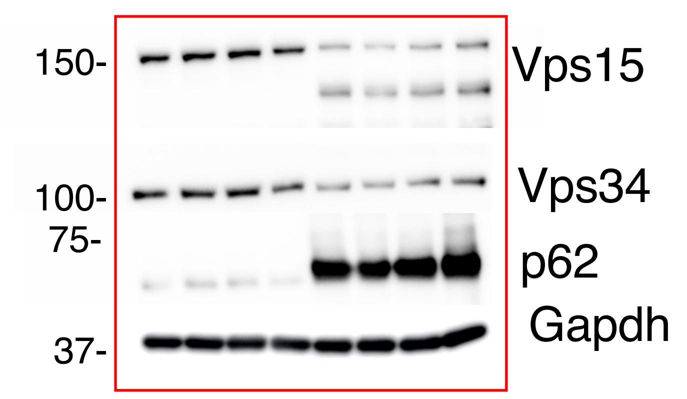


e.

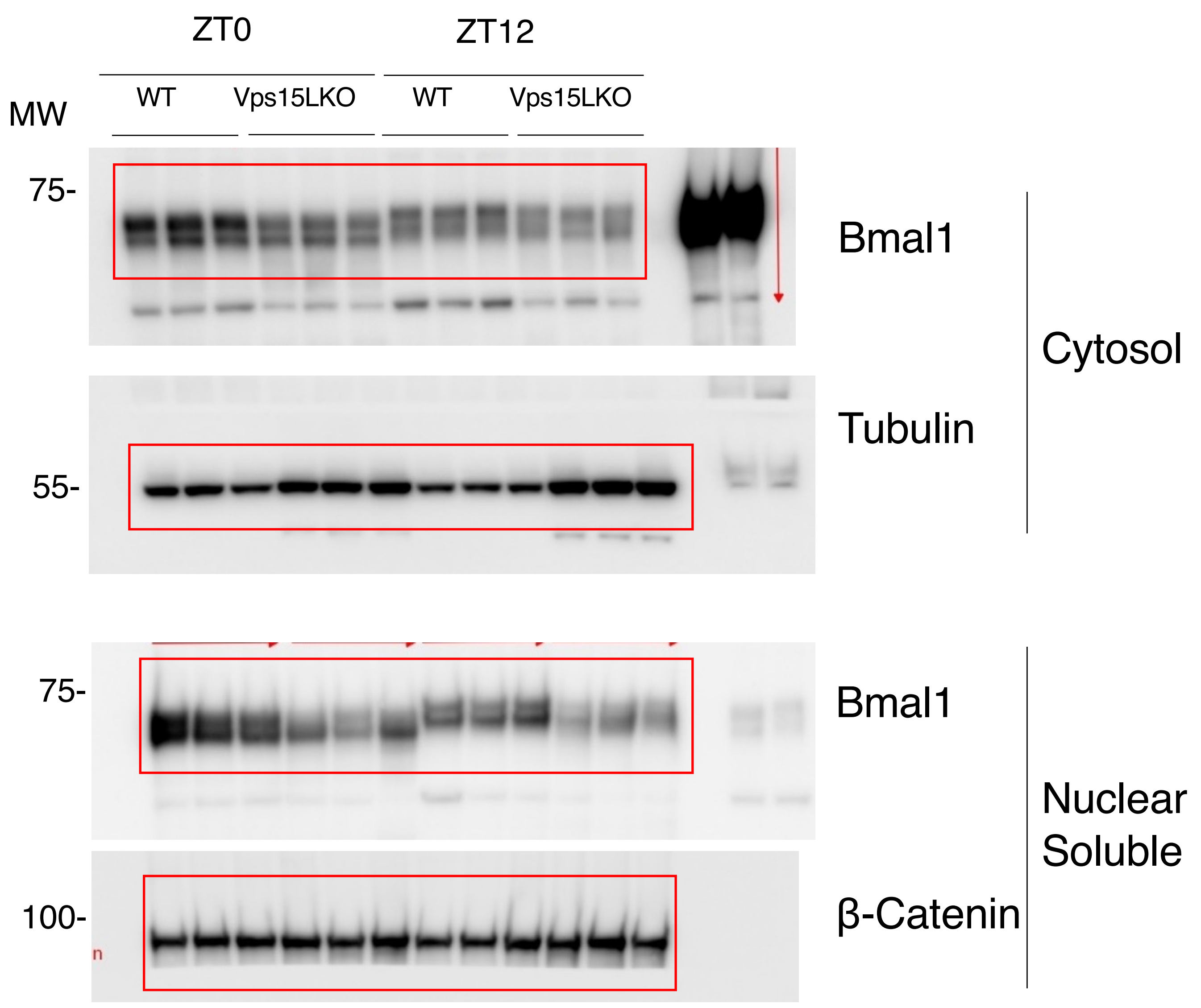


f.

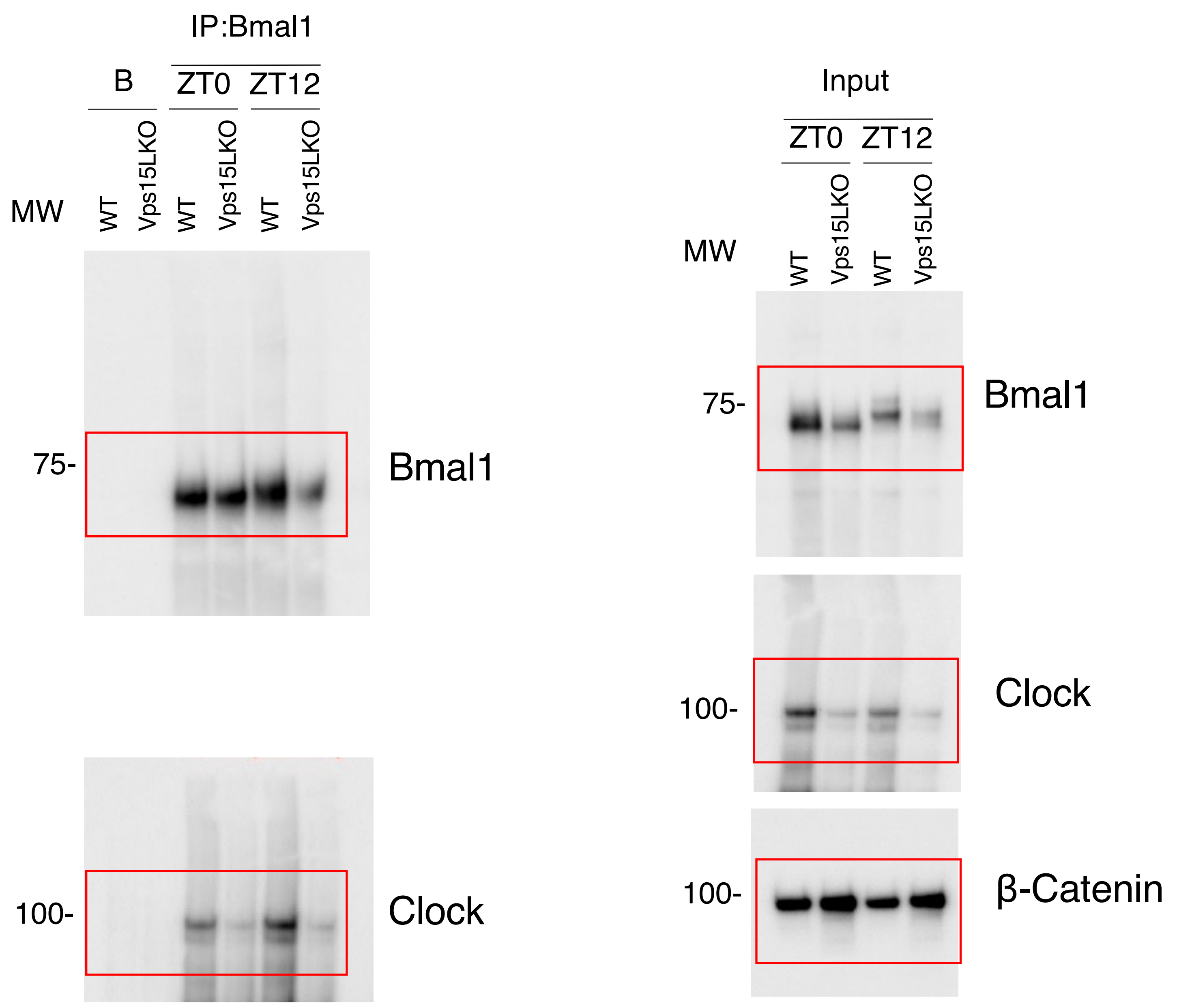

**Uncropped blots Extended Data Fig. 2.** Red square indicates the section shown in the main figures. Molecular weight is shown on the left.

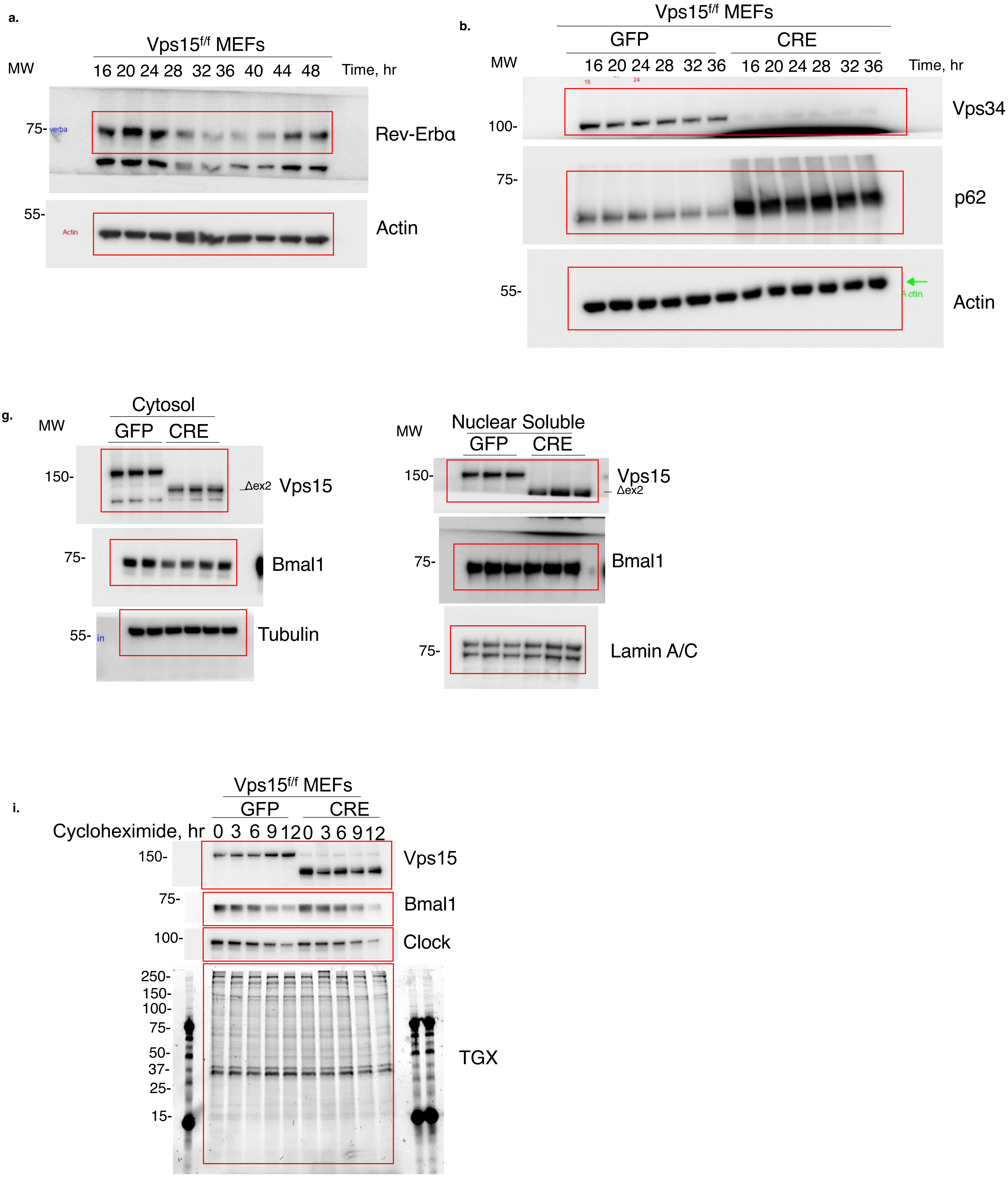

**Uncropped blots Extended Data Fig. 3.** Red square indicates the section shown in the main figures. Molecular weight is shown on the left.

a.

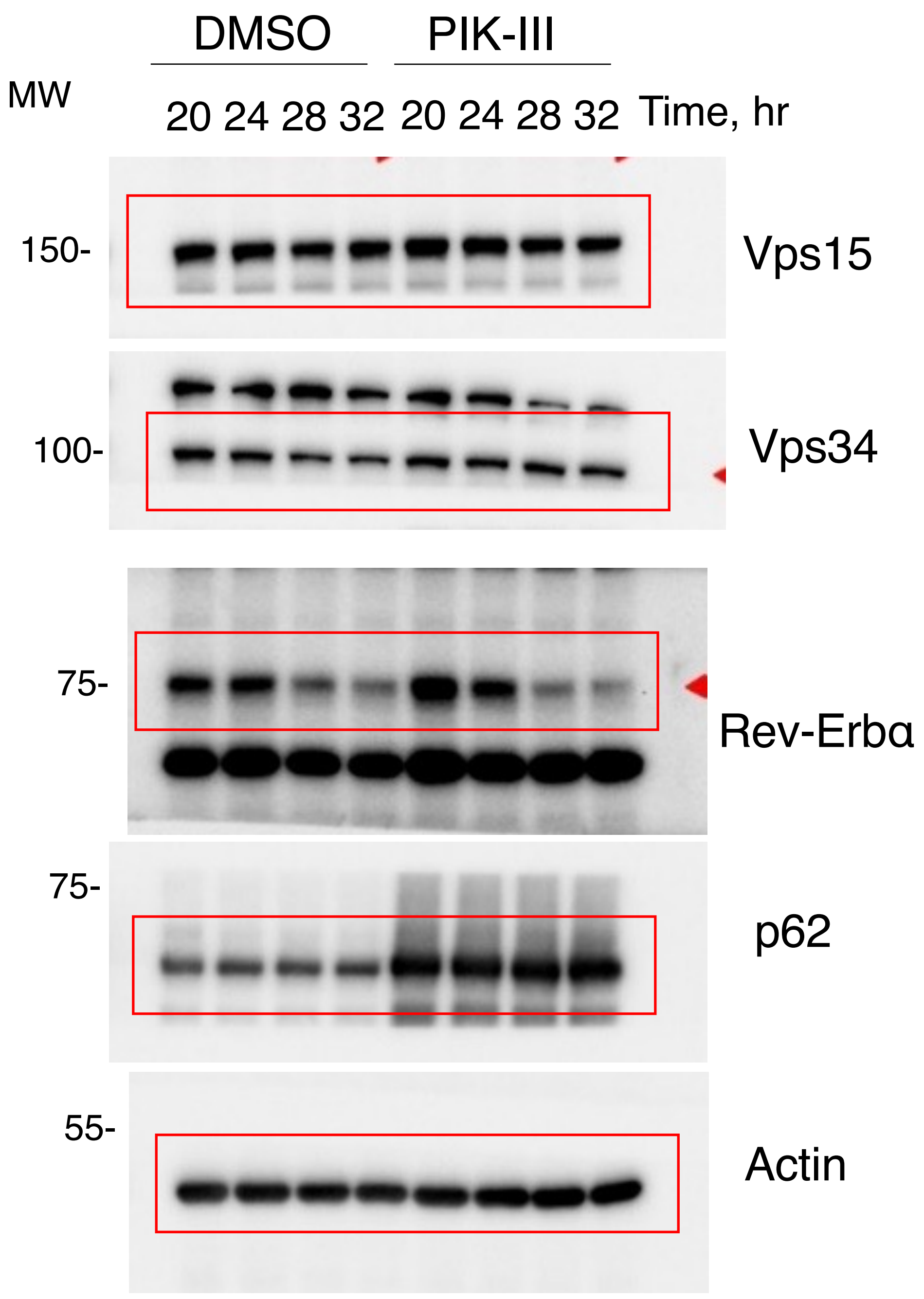


d.

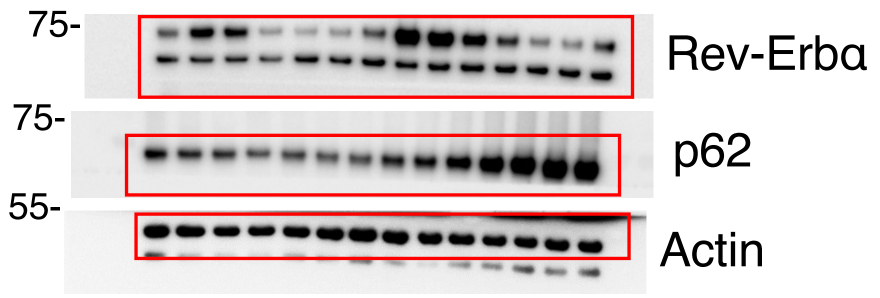


g.

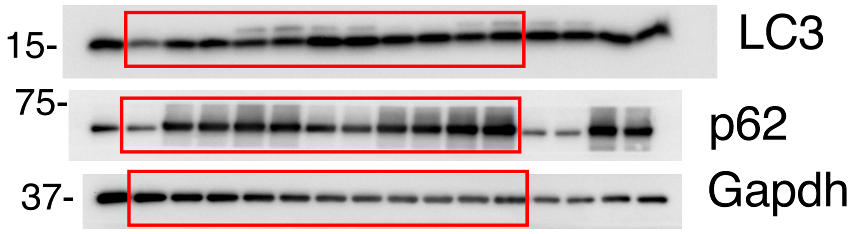

**Uncropped blots Extended Data Fig. 4.** Red square indicates the section shown in the main figures. Molecular weight is shown on the left.

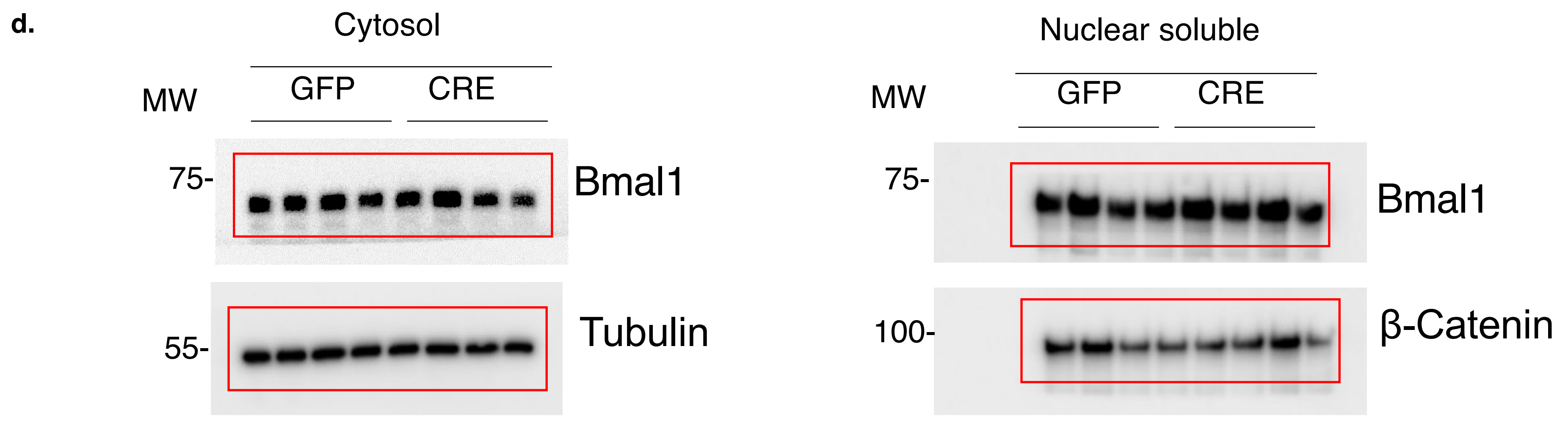

**Uncropped blots Extended Data Fig. 5.** Red square indicates the section shown in the main figures. Molecular weight is shown on the left.

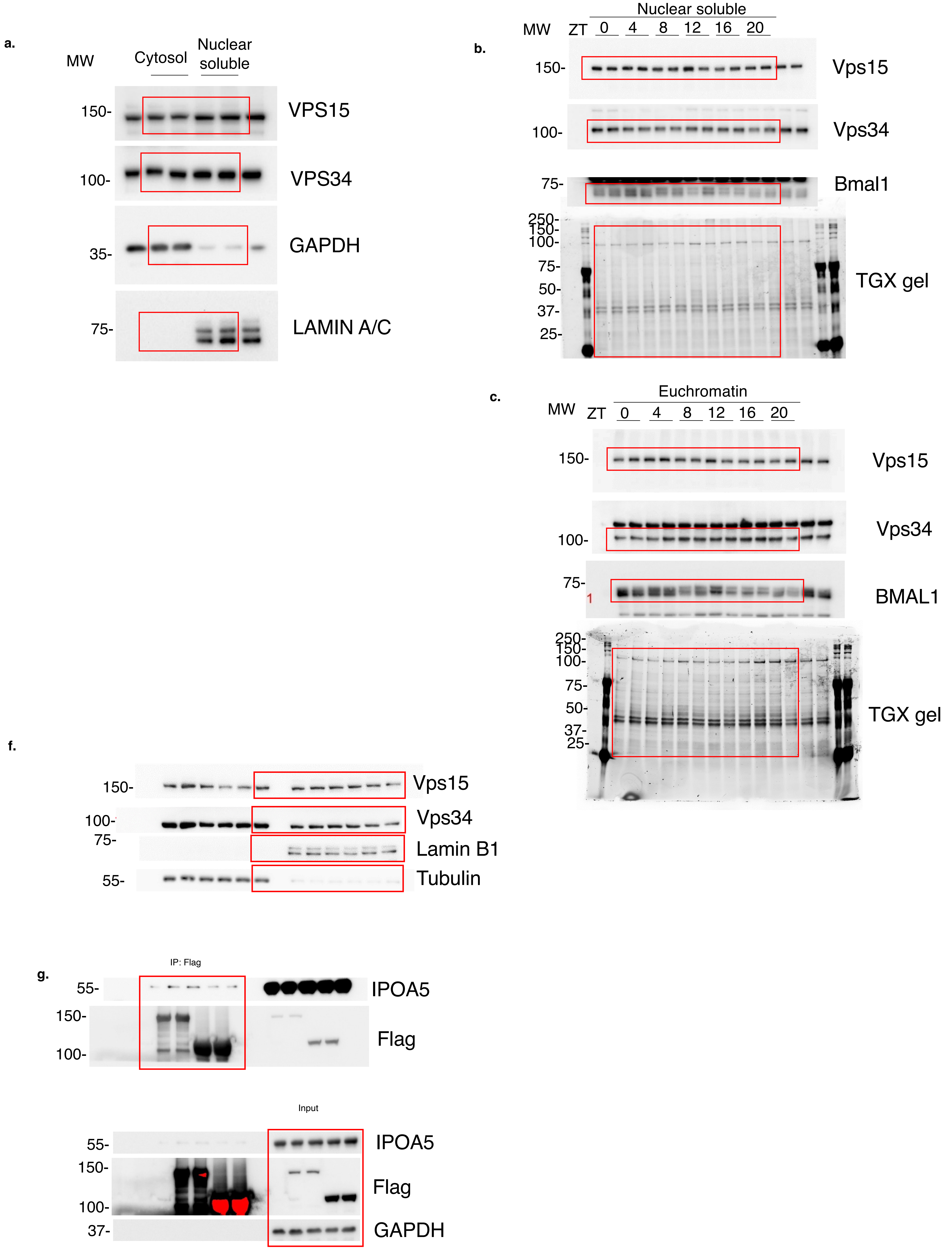

**Uncropped blots Extended Data Fig. 6.** Red square indicates the section shown in the main figures. Molecular weight is shown on the left.

**b.**

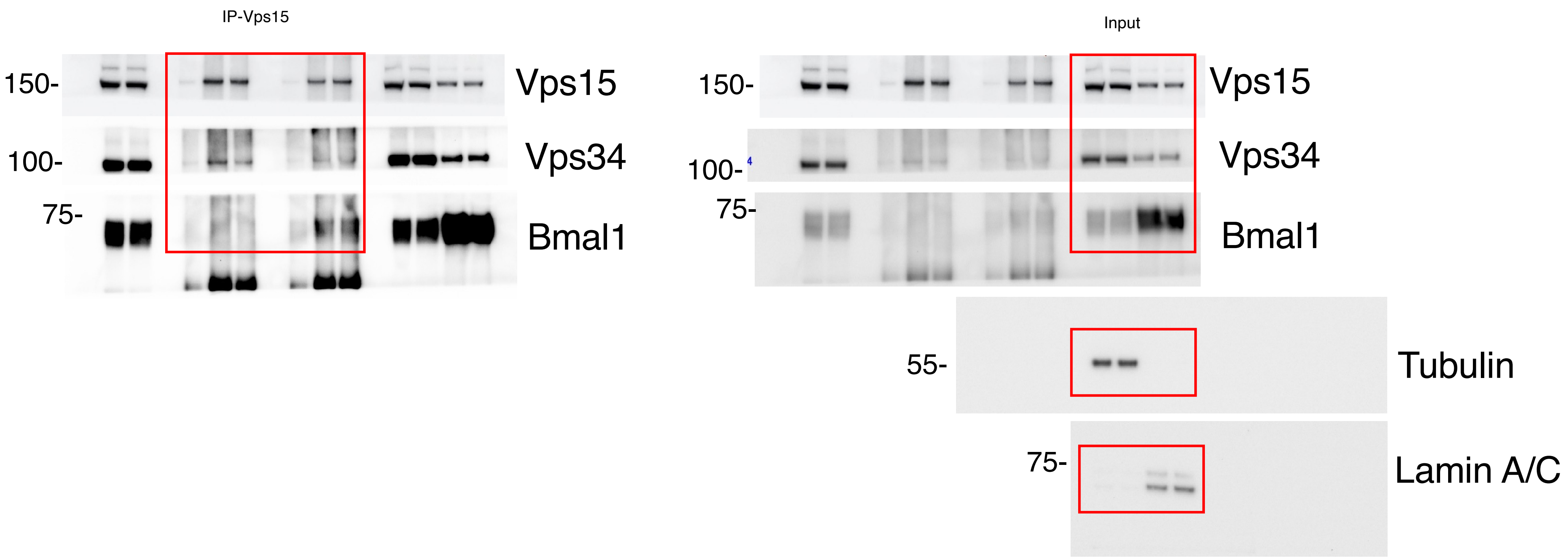


**e.**

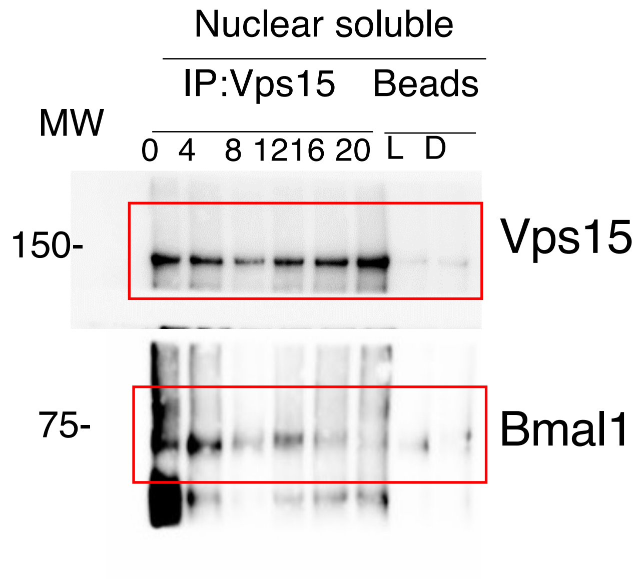


**f.**

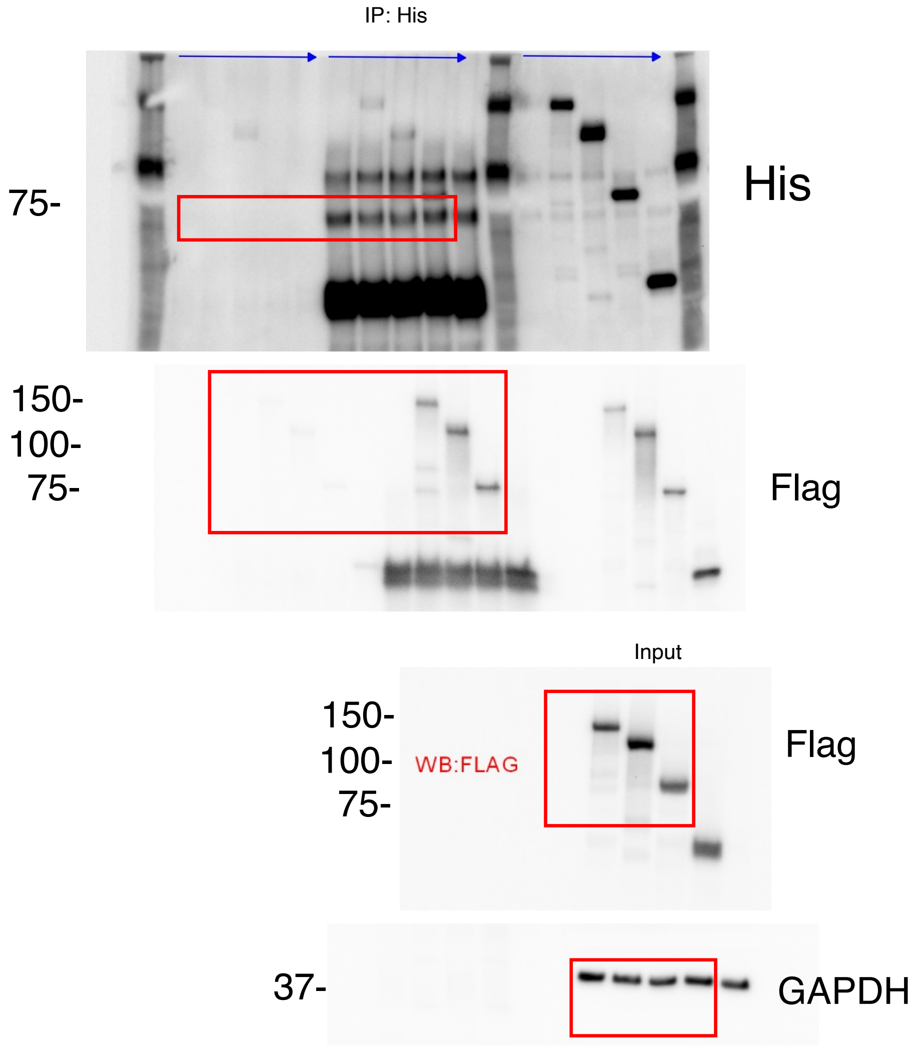

**Uncropped blots Extended Data Fig. 7.** Red square indicates the section shown in the main figures. Molecular weight is shown on the left.

b.

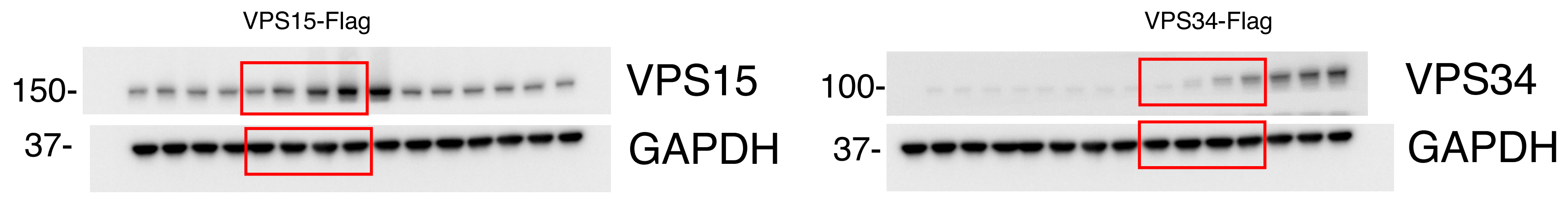


d.

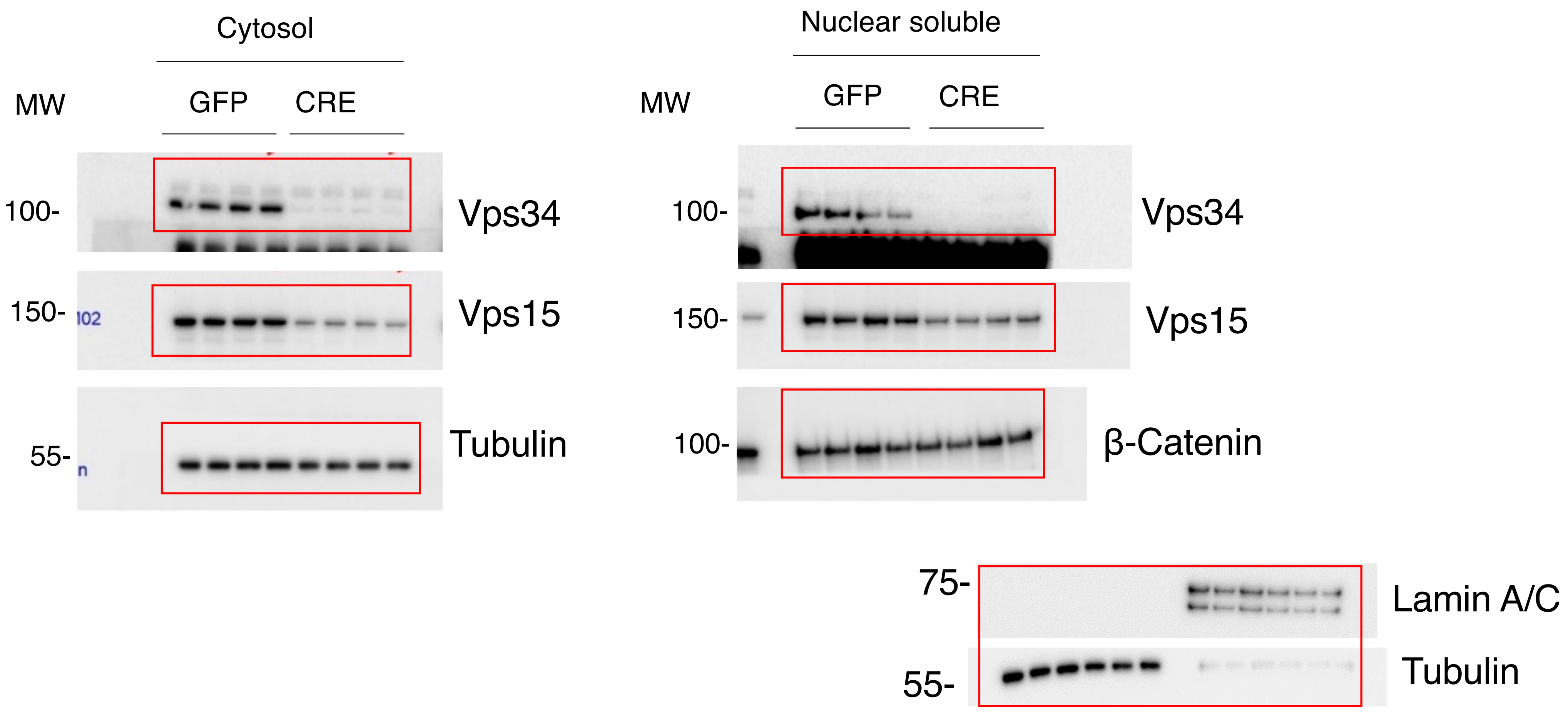


e.

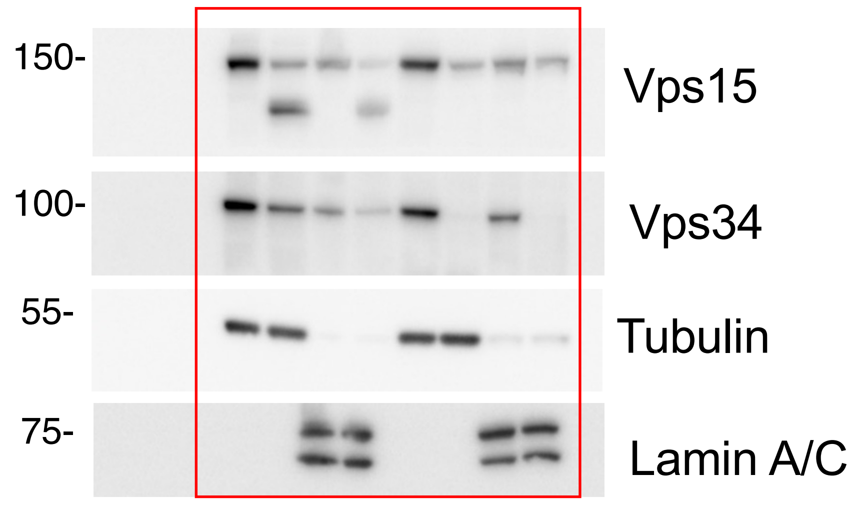

**Uncropped blots Extended Data Fig. 9.** Red square indicates the section shown in the main figures. Molecular weight is shown on the left.

a.

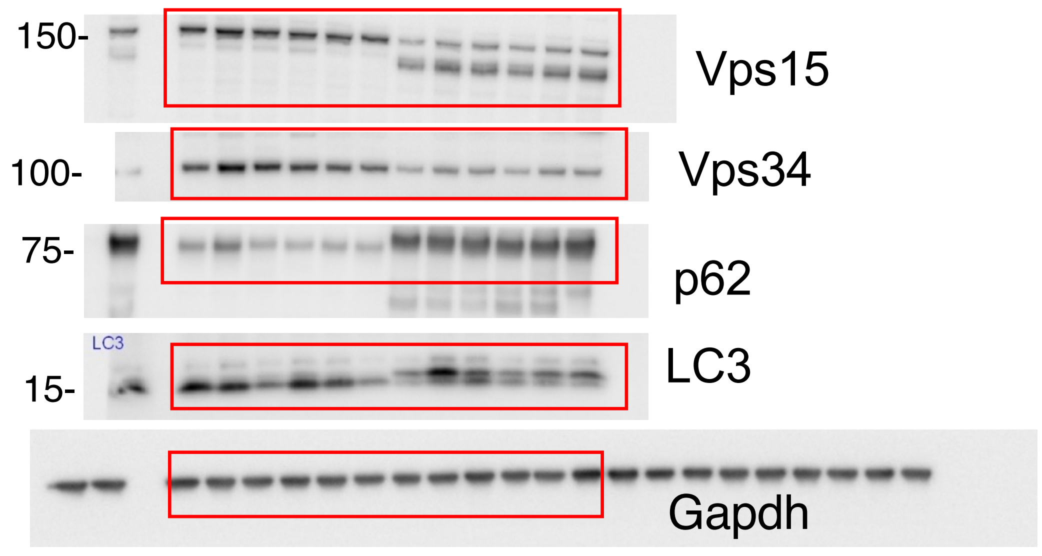


b.

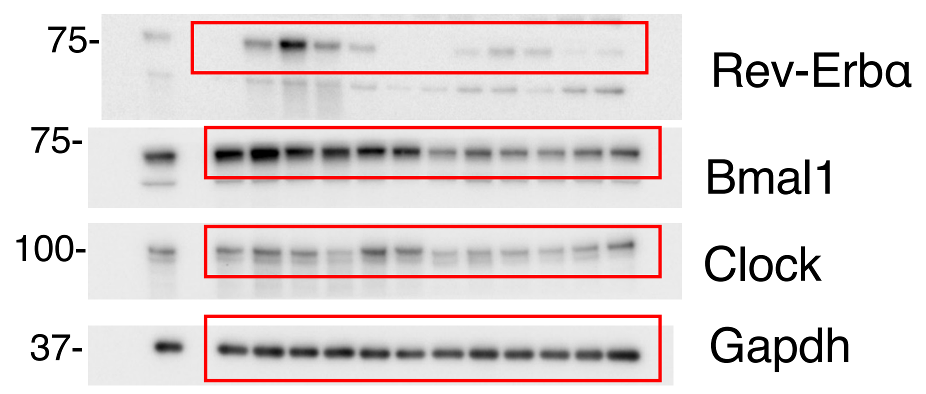


c.

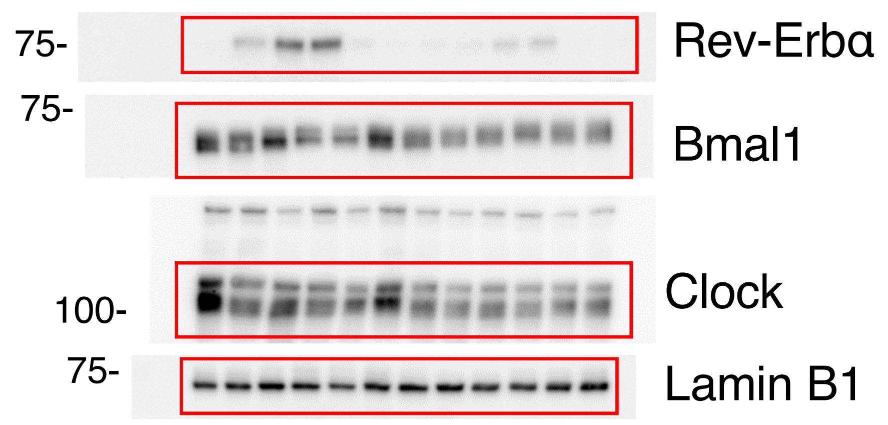

**Uncropped blots Extended Data Fig. 10.** Red square indicates the section shown in the main figures. Molecular weight is shown on the left.

**b.**

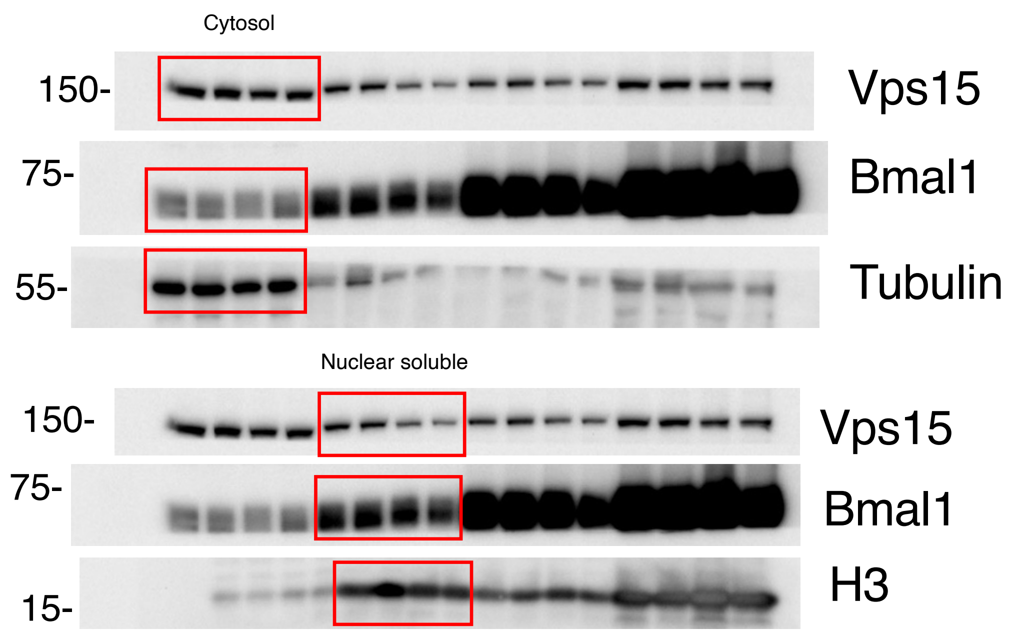

Supplement: Source Data — Uncropped western blots in Figs. 1–6 and Extended Data Figs. 1–7,9,10. [file 41556_2023_1171_MOESM6_ESM.pdf]
